# Adaptation to Impacts of Climate Change on Aeroallergens and Allergic Respiratory Diseases

**DOI:** 10.3390/ijerph7083006

**Published:** 2010-07-28

**Authors:** Paul J. Beggs

**Affiliations:** Environmental Science, Department of Environment and Geography, Faculty of Science, Macquarie University, Sydney, New South Wales 2109, Australia; E-Mail: paul.beggs@mq.edu.au; Tel.: +61-(0)2-9850-8399; Fax: +61-(0)2-9850-8420

**Keywords:** climate change, impacts, adaptation, human health, pollen, mould spore, allergen, allergic disease, asthma, allergic rhinitis

## Abstract

Climate change has the potential to have many significant impacts on aeroallergens such as pollen and mould spores, and therefore related diseases such as asthma and allergic rhinitis. This paper critically reviews this topic, with a focus on the potential adaptation measures that have been identified to date. These are aeroallergen monitoring; aeroallergen forecasting; allergenic plant management; planting practices and policies; urban/settlement planning; building design and heating, ventilating, and air-conditioning (HVAC); access to health care and medications; education; and research.

## 1. Introduction

Although research on the physical science and impacts of climate change will continue into the future, there is now a considerable body of research on the impacts of climate change on aeroallergens and allergic respiratory diseases, and adaptation responses to these impacts. Although this impacts research has been reviewed by several authors recently, an overview and critical review of the adaptation responses has not yet been conducted. This is the aim of this paper. In the following sections, climate change (both observed in the recent past and projected into the future) is described, followed by a summary of the potential impacts of these changes on aeroallergens and allergic respiratory diseases, with the latter placed in context through a synthesis of the research on the public health importance of these diseases. The major focus of the paper is a review of adaptation responses to these potential impacts, including: aeroallergen monitoring; aeroallergen forecasting; allergenic plant management; planting practices and policies; urban/settlement planning; building design and heating, ventilating, and air-conditioning (HVAC); access to health care and medications; education; and research.

## 2. Climate Change

The Intergovernmental Panel on Climate Change (IPCC) in its Fourth Assessment Report (AR4), the most recent, states that global atmospheric concentrations of greenhouse gases such as carbon dioxide, methane and nitrous oxide have increased markedly as a result of human activities since 1750, and that most of the observed increase in global average temperatures since the mid-20th century is very likely due to the observed increase in anthropogenic greenhouse gas concentrations [1]. The global atmospheric carbon dioxide concentration ([CO_2_]) has increased from a pre-industrial value of about 280 parts per million (ppm) to 386.30 ppm in 2009 [2]. The 100-year linear trend (1906–2005) in global average surface temperature is 0.74 °C (0.56 °C to 0.92 °C) [1].

Future increases in emissions and atmospheric concentrations of greenhouse gases, and resulting increases in surface temperature are projected [1]. Global atmospheric carbon dioxide concentration is projected to increase from its current level to anywhere between about 500 ppm and over 1,100 ppm by the year 2100 [3]. Global average surface warming is projected to range from 1.1 °C to 6.4 °C at 2,090–2,099 relative to 1,980–1,999, depending on which greenhouse gas emissions scenario is followed [1]. Changes in many other aspects of the climate system are also projected. These include increases in the amount of precipitation in high latitudes, and decreases in most subtropical land regions; more intense tropical cyclones (typhoons and hurricanes), with larger peak wind speeds and more heavy precipitation; and poleward movement of extratropical storm tracks, with consequent changes in wind, precipitation and temperature patterns [1].

## 3. Climate Change Impacts

Climate change impacts on many physical, biological and human systems. Although human health is clearly part of the latter system(s), many diseases are intimately related to the environment, and therefore impacts of climate change on physical and biological systems may also result, indirectly, in impacts on human health. Climate change has had, and will continue to have, many significant adverse impacts on human health. The impacts of climate change on allergic respiratory diseases such as asthma and allergic rhinitis, via impacts on aeroallergens such as pollen and mould spores, are now emerging as one of the major indirect impacts of climate change on public health. The impacts of climate change on aeroallergens were reviewed by Beggs [4] and more recently by Confalonieri *et al*. [5], and the resulting impacts on allergic respiratory diseases have been explored recently by Beggs and Bambrick [6] and Shea *et al*. [7]. Further, Schmier and Ebi [8] recently examined the impacts of climate change and aeroallergens on children’s health (specifically asthma). Plants, a source of many clinically important allergens, are particularly sensitive to climate change as a result of not only their response to changes in temperature, rainfall, and other climate variables, but also their response to changes in [CO_2_]. The impacts of climate change on aeroallergens, and in particular pollen, include impacts on pollen production and atmospheric pollen concentration, pollen season, plant and pollen spatial distribution, pollen allergenicity, and similar impacts on mould spores. Details of, and evidence for, these impacts is provided in the following sections.

### 3.1. Pollen Production and Atmospheric Pollen Concentration

There are two lines of evidence that suggest increases in [CO_2_] and temperature can result in increases in pollen production and atmospheric pollen concentrations. Experimental research where ragweed (*Ambrosia artemisiifolia*) has been grown at pre-industrial, current, and potential future [CO_2_]s has found that pollen production in this species is significantly increased at current compared to pre-industrial [CO_2_] and also at potential future compared to current [CO_2_] (e.g., Ziska and Caulfield [9]). Similar research, comparing pollen production in ragweed grown in the higher temperature and [CO_2_] of an urban environment to that grown in the adjacent rural environment, also found increased production in this species in the conditions of the urban environment: analogous to a future global atmosphere [10].

Atmospheric pollen concentrations have been monitored at many locations around the world, and records extending over one or more decades during the last half century or so exist for locations in Europe, Japan, and North America. Many of these records show an increasing trend in pollen concentration for important allergenic species such as birch (*Betula*) (e.g., Yli-Panula *et al*. [11]), alder (*Alnus*) (e.g., Bortenschlager and Bortenschlager [12]), and Japanese cedar (*Cryptomeria japonica*) [13]. In some cases, the increasing trend has been associated with increasing regional temperatures (e.g., Yli-Panula *et al*. [11]). These records therefore suggest that climate change may already be having an impact on atmospheric pollen concentrations.

### 3.2. Pollen Season

The extended monitoring of atmospheric pollen concentrations referred to above has also enabled changes in the timing and length of the pollen season to be examined. Such studies have generally found that the start of the pollen season is getting earlier. For example, van Vliet *et al*. [14] analysed daily pollen counts for 14 plant species or families from 1,969 to 2,000 in the western part of the Netherlands, and found an advance of the mean start of the pollen season of 3 to 22 days for the decade of the 1990s compared to the decade of the 1970s. The results also indicated that there was a strong correlation between temperature and the start of the pollen season. This finding of a trend to earlier pollen season starts is consistent with phenological research showing that timing of many other plant (and animal) phenomena, such as flowering, is also getting earlier in response to increasing atmospheric temperatures [15].

A clear trend in the timing of the end of the pollen season, and therefore the overall length of the pollen season, has not been found. Some studies have found that the end of the pollen season has either remained constant or got later, so when combined with an earlier start resulting in a longer overall pollen season [5], while other studies have found that just like the start of the pollen season, the end of the pollen season is occurring earlier, resulting in little or no overall change in the length of the pollen season [5]. In terms of projected future changes in the timing of the pollen season, several modelling studies indicate a continuation of the trend to an earlier pollen season [16].

### 3.3. Plant and Pollen Spatial Distribution

Relatively few studies have directly examined the impacts of climate change on the spatial distribution of allergenic plants or the dispersion of their pollen. However, based on the most recent assessment of the IPCC [17], that there is very high confidence that recent warming is resulting in poleward and upward shifts in ranges in plant species, such shifts are likely to be occurring in plant species that produce clinically important pollen. Emberlin [18] has suggested such shifts in the range of *Betula* in the Northern Hemisphere are likely with future climate change. For future increases in global average temperature exceeding 1.5–2.5 °C, there are projected to be major changes in species’ geographical ranges [17]. The implications of these changes are that pollen exposure may change over time, with the potential for a decrease or disappearance of some pollen types, and an increase or appearance of other pollen types.

In addition to these changes in plant ranges, changes in atmospheric circulation patterns may have an impact on the dispersion of pollen [18]. D’Amato *et al*. [19] have described the potential for changes in atmospheric circulation patterns to contribute to episodes of long distance transport of allergenic pollen. It has also been suggested that increasing air temperature may enhance atmospheric instability, thereby altering the turbulent airflow that transports pollen [20]. Climate change may also have an impact on allergic respiratory diseases through impacts of extreme events on aeroallergens, including thunderstorms and tropical cyclones [21].

### 3.4. Pollen Allergenicity

There may also be impacts of climate change on pollen allergenicity (here defined as the allergen content or concentration of the pollen grain). Research by Singer *et al*. [22] found that ragweed grown at a potential future [CO_2_] produced pollen that was significantly more allergenic than pollen produced by plants grown at both current and pre-industrial [CO_2_]. There is also some evidence to suggest that higher air temperature can increase birch pollen allergenicity [23,24]. This is supported by more-recent research by Tashpulatov *et al*. [25].

### 3.5. Mould Spores

Although the body of research on the impacts of climate change on mould spores is much smaller than that on pollen, there are several important studies that indicate similar impacts on mould spore production and atmospheric mould spore concentration, mould spore season, and mould spore allergenicity. Two of these aspects have just been studied experimentally by Wolf *et al*. [26] who found *Alternaria alternata* (a ubiquitous allergenic fungus) grown on timothy plants grown at potential future [CO_2_]s, produced nearly three times more spores and more than twice the total antigenic protein per plant than at lower [CO_2_]s. Earlier observational research by Corden and Millington [27] has also shown long-term trends of *Alternaria* mould spores in Derby, United Kingdom (UK). From the early 1970s to the late 1990s, spore concentrations increased and the spore season started earlier and increased in duration, trends that were associated with increases in local temperature.

## 4. Public Health Implications

Pollen and mould spore allergens are significant to human health. The prevalence of pollen allergy in asthmatics varies by location and age, with, for example, recent research finding 7% of a large sample of children with allergic disease in the Netherlands sensitised to grass pollen [28]; almost 40% of 1- to 3-year old children with asthma in a United States of America (US) sample showing IgE-mediated sensitivity to outdoor pollen allergens [29]; and another study suggesting “Pollen allergy has been found in 80–90% of childhood asthmatics and 40–50% of adult-onset asthmatics” [30], and pollen allergenic fragments are respirable and are likely correlated with the asthmatic response in allergic asthmatics [30]. The associated mechanisms and pathophysiology are complex and have been the subject of a growing body of literature. Common ragweed is the principal source of pollen associated with seasonal allergic rhinitis in the US, and is spreading in other parts of the world [5]. The results of the above mentioned experimental studies therefore have important public health implications. The clinical importance of *Alternaria* exposure in children in rural towns has been demonstrated by Downs *et al*. [31], who concluded that *Alternaria* allergens contribute to severe asthma in regions where exposure to the fungus is high. Consistent with this is the research suggesting that exposure to environmental moulds may play a role in asthma-related mortality [32].

Asthma is the most common chronic disease among children, with recent “International Study of Asthma and Allergies in Childhood” results showing the global total prevalence of asthma symptoms in the past 12 months (current wheeze) in 13–14 year olds at 14.1%, with the prevalence in some locations more than double this [33]. Australia is one such location, and it is research from there (and elsewhere) that has also shown that at least some indigenous populations may be particularly vulnerable to the impacts of climate change on this disease. For example, research suggests that Australian Aboriginal and Torres Strait Islander children have more recent wheeze and parent-reported asthma than non-indigenous children [34]; and that the risk of reattendance at hospital for asthma within 28 days of an initial attendance is significantly higher among Australian indigenous people [35]. Asthma is also an important contributor to the burden of impaired quality of life. Again, as an example, recent research in the Australian community has found the presence of asthma in 18–64 year olds accounts for relatively large percentages of the people reporting poor life satisfaction, poor health status, high psychological distress, and any reduced activity days [36].

Allergic rhinoconjunctivitis is another disease likely to be impacted by future changes in aeroallergens. Recent international research on Europe, the US, Australia and New Zealand, shows that the prevalence of this disease, at least in adults, is even higher than that for asthma, with 11.8–46.0% (and median 24.8%) of 20–44 year olds with nasal allergy [37]. Allergic rhinoconjunctivitis, like asthma, has substantial impacts on quality of life and considerable economic and societal burden. For example, a recent review of published data pertaining to the impact of rhinitis on work disability showed that while there was a rather modest effect on absenteeism; rhinitis is associated with impairment in at-work performance and lost productivity attributable to reduced on-the-job effectiveness of 11–40% [38]. Finally, while not a focus of this review, it is important to note that aeroallergens such as pollen are now being associated with other important diseases such as stroke [39].

Perhaps the most recent and most profound assessment of and response to this issue comes from the US Environmental Protection Agency’s (EPA’s) “Endangerment and Cause or Contribute Findings for Greenhouse Gases under the Clean Air Act” of 7 December 2009. As one of only seven key health effects that support EPA’s determination that current and future concentrations of greenhouse gases endanger public health, is that “Climate change could impact the production, distribution, dispersion and allergenicity of aeroallergens and the growth and distribution of weeds, grasses, and trees that produce them. These changes in aeroallergens and subsequent human exposures could affect the prevalence and severity of allergy symptoms” [40]. The impacts of climate change on pollen and mould spores described above are therefore an important public health concern that requires development and implementation of appropriate response strategies now.

## 5. Adaptation

From a climate change perspective, two basic responses to the potential impacts on aeroallergens and allergic respiratory diseases exist: mitigation and adaptation. Mitigation, as defined by the IPCC, is “An anthropogenic intervention to reduce the anthropogenic forcing of the climate system; it includes strategies to reduce greenhouse gas sources and emissions and enhancing greenhouse gas sinks” [41]. While mitigation efforts must continue, adaptation is also required as a result of inevitable climate change. Whereas mitigation focuses on greenhouse gas concentration reductions, adaptation in the context of climate change is simply defined as “Adjustment in natural or human systems in response to actual or expected climatic stimuli or their effects, which moderates harm or exploits beneficial opportunities” [41].

Shea *et al*. [7] have recently expressed climate change adaptation and mitigation from a clinical perspective, stating that “For clinicians caring for atopic patients and patients with asthma, … both adaptation to disease linked to inevitable climate change (secondary prevention) and mitigation of the drivers that will worsen climate change (primary prevention) are important strategies to employ to minimize disease burden”. Indeed, one of the intentions of the review by Shea *et al*. [7] is to “stimulate action on the part of health care providers to become involved in adaptation and mitigation strategies to help minimize the ultimate disease burden related to climate change”. Similarly, Ayres *et al*. [42] have stated that public health professionals, like others in the health and medical system, have an advocacy role in persuading governments at all levels around the world to maintain awareness and appropriate actions with respect to climate change.

There are likely to be both limits and barriers or challenges to adaptation as a response to climate change [43]. These include “physical and ecological”, and technological limits; and financial, “informational and cognitive”, and “social and cultural” barriers [43]. Adger *et al*. [44] have recently expanded on the social limits to adaptation to climate change, concluding that the issues of values and ethics, risk, knowledge and culture construct societal limits to adaptation, but that these limits are mutable. There are challenges of designing and implementing adaptation measures, including the potential complexity of such measures. There needs to be full consideration of an adaptation response before implementation. For example, opportunities for co-benefits as well as the potential for unintended adverse consequences should be considered. Assessment of climate change adaptation will continue over the coming years and decades, with, for example, the IPCC’s Working Group II contribution to the Fifth Assessment Report (AR5), due for publication in 2014, to include chapters on “Adaptation needs and options”, “Adaptation planning and implementation”, “Adaptation opportunities, constraints, and limits”, “Economics of adaptation”, and “Climate-resilient pathways: adaptation, mitigation, and sustainable development” [45].

Perhaps a reflection of the relative infancy of the climate change adaptation field, some diversity in adaptation terminology exists. In particular, the literature refers to adaptation responses, adaptation strategies, adaptation options, adaptation measures, adaptation policies, and sometimes simply just adaptations. While a full assessment of each of these terms is not the purpose of this review, some clarification is justified here. Climate change adaptation “policies, generally speaking, refer to objectives, together with the means of implementation. In an adaptation context, a policy objective might be drawn from the overall policy goals of a country” [46] or for the purposes of this paper, sector. Climate change adaptation “Measures can be individual interventions or they can consist of packages of related measures. Specific measures might include actions that promote the chosen policy direction” [46]. And finally, a climate change adaptation “Strategy—refers to a broad plan of action that is implemented through policies and measures. A climate change adaptation strategy for a country refers to a general plan of action for addressing the impacts of climate change, including climate variability and extremes. It may include a mix of policies and measures, selected to meet the overarching objective of reducing the country’s vulnerability. Depending on the circumstances, the strategy can be comprehensive at a national level, addressing adaptation across sectors, regions and vulnerable populations, or it can be more limited, focusing on just one or two sectors or regions” [46].

Markandya and Chiabai [47] have recently examined climate change impacts on human health, including classification of these impacts into direct and indirect, and consideration of these impacts by region and development status. The major focus of the research, however, is the investigation of the costs of climate change impacts on human health and the costs of planned adaptation in the health context. While this is an important contribution to our understanding of adaptation for impacts of climate change on human health, and a wide range of diseases and health impacts are considered, there is no mention of the impacts of climate change on aeroallergens and allergic respiratory diseases.

The following sections review the research on adaptation for the potential impacts of climate change on aeroallergens and allergic respiratory diseases. Nine categories of adaptation in this area have been identified: aeroallergen monitoring; aeroallergen forecasting; allergenic plant management; planting practices and policies; urban/settlement planning; building design and HVAC; access to health care and medications; education; and research. These are examined in turn.

### 5.1. Aeroallergen Monitoring

Several authors have emphasised the need for improved surveillance or monitoring of atmospheric pollen and mould spore concentrations [21,48,49]. For example, English *et al*. [48] have recently identified pollen as one of only six environmental health indicators of climate change for the US, stating that the spatial coverage of the pollen-monitoring stations in the US is sparse, and that it would be preferable to increase the number of pollen-monitoring stations. This is significant given the relatively well-established network that currently exists in the US. Specifically, the American Academy of Allergy, Asthma and Immunology’s (AAAAI’s) Aeroallergen Network and National Allergy Bureau™ (NAB™) (that is responsible for reporting current pollen and mould spore levels to the public) currently has no less than approximately 78 counting stations throughout the US, two counting stations in Canada, and two counting stations in Argentina [50]. Similarly, the recent European Respiratory Society position statement on climate change and respiratory disease, which identifies gaps in knowledge and recommendations for research, includes the need for systems to be put in place to monitor changes in aeroallergen concentrations [42]. Again, this is with existing well-established networks such as the European Aeroallergen Network (EAN) “polleninfo.org”, and atmospheric allergen quantification techniques such as those currently being trialled in the UK and Europe through the European Union “Health Impacts of Airborne Allergen Information Network” (HIALINE) Project.

English *et al*. [48] also state that “To obtain more complete coverage of pollen levels for the United States, either modeling or the use of satellite imagery to generate detailed land use coverage (to project the distribution of ragweed) would be necessary”. With respect to aeroallergen observation, remote sensing such as the use of satellite imagery is very much an emerging technology. Integration and coordination of ground and space-based (and possibly other) observation instruments, systems and networks will become increasingly important, with assessments such as the current Group on Earth Observations (GEO) Task US-09-01a to identify critical earth observation priorities for Societal Benefit Areas (one of which is Human Health through Aeroallergens) [51] a vital step towards this. However, English *et al*. [48] note that more complete coverage of pollen levels via remote sensing “would not provide real-time airborne pollen data, so it would be preferable to increase the number of pollen-monitoring stations”.

Although there is relatively good aeroallergen monitoring in some parts of the world, in others such monitoring is sparse or absent. Enhanced aeroallergen monitoring is particularly required in the latter areas, including in many rural locations.

### 5.2. Aeroallergen Forecasting

Similarly, aeroallergen forecasting currently occurs in some locations, but is limited or absent in many other locations around the world. Enhancement of such forecasting will enable both individuals with allergic respiratory diseases and their carers (parents, teachers, and health professionals) in more locations to better manage within-season variability of aeroallergen levels. Sofiev *et al*. [52] have recently examined, in depth, plant-induced human allergy and adaptation to impacts of climate change on it via short-to-mid-term forecasts of atmospheric pollen concentrations and following pre-emptive and preparatory measures. Local aeroallergen monitoring and forecasting potential is required so that management and prevention of allergic respiratory symptoms can occur, otherwise there exists the danger that increased adverse health impacts will result into the future.

### 5.3. Allergenic Plant Management

Allergenic plant management is used effectively in some countries as a defence strategy against important allergenic plants and the diseases with which they are associated. For example, Rybníček and Jäger [53] have documented government-led eradication campaigns for ragweed in Europe. Therefore, another adaptive measure would be tighter management of allergenic plant species.

### 5.4. Planting Practices and Policies

Government authorities could consider more carefully which plant species are used in populated areas [4]. Species selection is also important to avoid aeroallergen from climate change mitigation tree planting and urban reforestation. Younger *et al*. [54] have examined the nexus between the built environment, public health, and climate change mitigation and adaptation, stating that “Working across sectors to incorporate a health promotion approach in the design and development of built environment components may mitigate climate change, promote adaptation, and improve public health”. As an example of this, they list specific improved land-use planning approaches for reducing greenhouse gas emissions, including “creating new green spaces (e.g., on roofs and along streets and railroad lines), … maintaining existing green spaces, conserving natural lands through controlled development, and planting trees with high growth rates for additional green cover”. The aeroallergen potential of all the species to be used in these approaches must be carefully considered. “Similarly, private individuals could transform their gardens into low allergen gardens by planting low or non allergenic species” [21]. Plants that are pollinated by birds and insects rather than wind are preferable for the purposes of aeroallergen exposure reduction.

The intended outcome of better allergenic plant management and planting practices and policies would be a long-term reduction in the ambient pollen allergen concentration, which would contribute to a reduction of allergic respiratory disease burden.

### 5.5. Urban/Settlement Planning

There are aspects of urban or settlement planning that can play a role in the link between climate, aeroallergens and allergic respiratory disease. Greater consideration of these aspects is therefore another way in which to adapt to the potential impacts of climate change on aeroallergens and related diseases. For example, “Greater awareness of the impacts of indoor moisture on molds and associated respiratory diseases should provide additional incentive to shift housing development away from flood-prone areas” [49].

One of the challenges of the planting practices and policies and urban/settlement planning adaptation measures described above will be to ensure that a related adaptation measure for another important human health impact of climate change is compatible. Schmidt [55], for example, has stated that one adaptation measure targeting human health (specifically heat-related disease) is “optimal land use designs—such as more use of undeveloped “green space” and shade trees—to help keep city-dwellers cool”. While such undeveloped “green space” and shade trees are to be encouraged, the plant species used for these should be selected to minimise aeroallergen concentrations.

The opportunity exists to design or modify communities such that they promote overall health by increasing opportunities for physical activity. Increased physical activity potentially reduces the risk of being obese or overweight and related risk of type II diabetes—which are increasingly recognised as risk factors for onset or exacerbation of childhood and adult onset asthma (and allergic airways disease) [56].

### 5.6. Building Design and HVAC

There is a large body of literature and knowledge about healthy buildings. Building design and heating, ventilating and air-conditioning can be used to control both indoor allergen production and exchange of allergens between the indoor and outdoor environments (e.g., Fisk *et al*. 2002 [57]; Mendell *et al*. 2008 [58]). For the purposes of climate change adaptation, Kinney [49] has stated that “Use of innovative air handling and filtration equipment for reducing the penetration of outdoor pollens into indoor spaces may also be valuable”. Aeroallergens produced outdoors can also be carried indoors via shoes, clothes, and in pet furs *etc.* [59,60]. Washing clothing with water and a detergent eliminates a majority of the pollen from the fabric [59], and leaving shoes at the entry to buildings would reduce the spread of aeroallergens on them indoors.

Indoor aeroallergen sources include indoor mould, house dust mites, and allergens from cockroaches, rodents, and other pests. Although the impacts of climate change on indoor aeroallergens have received far less attention than those on outdoor aeroallergens, a recent report [61] has focused on this topic as part of a broader examination of the impacts of climate change on indoor environments and resulting public health consequences. The report highlights the issue of climate change, moisture and indoor mould, stating that: “Increased relative humidity from climate change will increase the moisture content of materials indoors and thus increase the risk for mold growth. These conditions will be exacerbated by heavy periodic rainfalls that will likely stress the ability of buildings of all types to adequately manage excess water flow” [61]. Associated adaptation measures suggested in the report are that “A careful analysis of regional vulnerabilities to moisture intrusion into existing buildings, and to building practices to prevent such intrusions in new building construction, would be worthwhile. In addition, widespread dissemination of guidelines for remediating dampness and mold in buildings, integrated pest management techniques, and revised specifications for temporary housing could help mitigate moisture-related public health consequences of climate change in buildings” [61].

### 5.7. Access to Health Care and Medications

Access to appropriate health care and medications is central to the management of allergic respiratory diseases such as asthma. For example, every asthmatic should have a written Asthma Action Plan prepared in partnership with their doctor that assists them to manage their asthma at different times by recognising changes in their symptoms and knowing what action to take in response to those changes in symptoms. In the case of aeroallergens, ensuring complete and equitable access to available medications will be increasingly important with climate change [49].

### 5.8. Education

Many aspects of allergic respiratory disease education may become increasingly important into the future. In the case of aeroallergens, stronger education programs directed at allergen avoidance will be important [49]. The trend to earlier pollen season starts should also be clearly communicated to those with allergic respiratory disease and their carers and related health care professionals.

### 5.9. Research

Continued research is required. This includes further climate model studies of future changes in thunderstorms, which can lead to short-term outbreaks of asthma symptoms [21]. Continuation of research involving long-term aeroallergen monitoring is particularly important, so that future trends in aeroallergen concentrations and seasonality are monitored. Much more experimental research on impacts of elevated [CO_2_] and temperature on pollen and allergen production of more plant species is required. Similarly, modelling and surveillance of future changes in allergenic plant ranges is required.

Finally, more research into adaptation in this area is required [21]. This is because knowledge of adaptation strategies in many systems and sectors, including human health, and regions, is relatively limited. This is particularly the case for adaptation strategies specific to the potential adverse impacts of climate change on aeroallergens and allergic respiratory diseases. As such, adaptation is a particularly active area of climate change research at present, and this is likely to continue into the future.

## 6. Conclusions

Climate change has the potential to impact on aeroallergens such as pollen and mould spores, including increases in production and atmospheric concentrations, earlier pollen and mould spore season starts, increased allergenicity, and changes in at least plant and pollen spatial distribution. These biological impacts of climate change will likely have significant adverse impacts on allergic respiratory diseases such as asthma and allergic rhinitis unless appropriate adaptation measures are implemented. A range of such adaptation measures have now been identified. The outcomes of some of these measures, such as modified planting practices and policies, would not eventuate for many years, and they should therefore be implemented as soon as possible.
